# Nondestructive Evaluation of Tensile Stress-loaded GFRPs Using the Magnetic Recording Method

**DOI:** 10.3390/ma17010262

**Published:** 2024-01-04

**Authors:** Ryszard D. Łukaszuk, Tomasz Chady, Marek J. Żwir, Krzysztof Gorący

**Affiliations:** 1 Doctoral School, West Pomeranian University of Technology, 70-313 Szczecin, Poland; ryszard.lukaszuk@zut.edu.pl; 2 Faculty of Electrical Engineering, West Pomeranian University of Technology, 70-313 Szczecin, Poland; 3 Department of Polymer and Biomaterials Science, Faculty of Chemical Technology and Engineering, West Pomeranian University of Technology, 70-311 Szczecin, Poland; marek.zwir@zut.edu.pl (M.J.Ż.); krzysztof.goracy@zut.edu.pl (K.G.)

**Keywords:** nondestructive testing, NDT, magnetic recording method, MRM, glass fiber-reinforced polymers, GFRP, tensile stress, residual magnetization, magnetic measurements

## Abstract

This paper presents the results of inspecting tensile stress-loaded GFRP (glass fiber-reinforced polymer) samples using the Magnetic Recording Method (MRM). The MRM can be utilized solely to examine ferromagnetic materials. The modification was proposed in order to examine nonmagnetic composites. Ferromagnetic strips made of low-carbon steel DC01 were bonded to the surface using an adhesive composed of epoxy resin with the addition of triethylenetetramine. The modified method’s feasibility was tested on six samples made of GFRP. The research procedure consisted of three steps. In the first step, a metal strip is glued at the top surface of each sample, and an array of 100 cylindrical permanent magnets is used to record a sinusoidal magnetic pattern on the strip. The initial residual magnetization is measured in the second step, and the samples are subjected to static stress. In the third step, the residual magnetization is measured one more time. Ultimately, the measurement results from the second and third steps are compared. Generally, the applied stress causes changes in the amplitude and frequency of the sinusoidal magnetization pattern. In the case of GFRP, the frequency changes have not been used for evaluation due to minimal variations. The statistical parameters (mean, median, max, and mode) of the RMS (root mean square) value of the sinusoidal pattern were calculated and analyzed. The analysis demonstrates that the modified method is suitable for providing unequivocal and exact information on the load applied to a nonmagnetic composite material. For the presented results, the applied load can be assessed unambiguously for the samples elongated up to 0.6%.

## 1. Introduction

Composite materials, especially those based on high-molecular-weight polymers, form an incredibly diverse set of construction, and often functional, materials [1,2,3,4]. This collection includes materials useful in both unique and typical applications. Composites are used in miniature and large-scale devices and all terrestrial environments and space. This array of applications is directly related to the great diversity of composite material properties. The reported tensile strength of glass fiber-reinforced composite materials ranges from 483 MPa to 4580 MPa, with an elastic modulus in the range of 35–86 GPa and an elongation at break of 1.2–5% [5]. A detailed characterization of the material used to assess the suitability of the MRM in monitoring the deformations of composites is provided in Section 2.

The functional characteristics of composites are designed and obtained during the manufacturing process according to needs—by selecting matrices and modifying phases, shaping the morphology and orientation of these phases, developing interfacial surfaces, generating interfaces, and engineering interactions at phase boundaries [6,7]. For composite materials, it is typical that the composite material product and the structural material are produced in the same technological process. Due to various simplifications and technical and economic limitations, the used composite material is not always fully optimized in terms of operational requirements in a specific application [8]. Usually, not all possible combinations of environmental interactions are evaluated, such as their impact on durability and changes in material properties. The development of composites, especially those performing critical functions in construction, is associated with extensive research into the properties of materials, usually destructive [9]. Such research includes specialized and most often standardized tests and measurements, each serving to obtain data on a specific material property—such as destructive deformation, modulus of elasticity, susceptibility to the action of a corrosive agent, and similar [9,10].

Nondestructive testing (NDT) plays a pivotal role in the modern world. Ensuring the high quality of manufactured components and their safe use is indispensable. A significant advantage of the NDT is that it does not affect the structural integrity of the tested material in any way. The increase in the need to perform NDT goes hand in hand with the continuous development of more advanced composite materials and stringent safety and environmental requirements [11]. It is essential to detect, localize, and identify inhomogeneities at the early stage of their formation to avoid failures that could compromise human lives and result in tremendous costs [12].

During the operational phase, the structure and structural material are monitored to detect signs of wear or the premature occurrence of dangerous states. Nondestructive testing methods are usually applied, starting from the simplest, such as organoleptic inspections, through various defectoscopy techniques (e.g., magnetic, X-ray, ultrasonic, microwave), deformation measurements, analyses of spontaneous emissions from the tested object (e.g., acoustic emission) or emissions induced by an agent with an intensity that does not pose a threat of damage to the object—mechanical, electrical, magnetic, or radiative [6,13].

In more detail, visual testing (VT) involves using the human eye and additional apparatus such as lenses, binoculars, or cameras to detect apparent surface structure damage. Several methods are feasible for detecting subsurface abnormalities. Impact Echo Testing (IET) uses the observation of mechanical or stress wave propagation inside the material. Tap testing (TT) relies on tapping the component surface with a hammer or other item and listening to the variations in the frequency of the resultant sound wave. Microwave testing (MT), ultrasonic testing (UT), and ground-penetrating radar (GPR) benefit from analyzing amplitude, phase, and frequency changes between the wave transmitted through the material and the received one. Infrared thermography (IRT) employs cameras to capture temperature changes between defective and unflawed areas of the sample. Laser testing methods (LT), e.g., shearography, include illuminating the material under testing with a laser, subjecting the material to external tensile or thermal stress, and registering both the initial and stress-induced image of the material. Radiography (RT) is based on passing X- or gamma-rays through a tested component and recording the transmitted wave intensity. Magnetic flux leakage (MFL) relies on detecting the leaked magnetic flux in ferromagnetic structures. Acoustic emission (AE) aims to register sound waves originating from the growth or propagation of internal defects [14].

In recent times, the possibility of employing ground-penetrating radar (GPR) and phased array ultrasonic testing (PAU) techniques for the detection of fiber reinforcement bars (FRP) embedded in concrete has also been considered. For example, a study by Malla et al. investigated concrete slabs with carbon, glass, and basalt bars. It was found that commercially available GPR devices can detect FRP, given that a higher penetrating wave frequency is applied. Regarding PAU, detecting glass fibers is unsatisfying, while localizing steel bars produces satisfactory results [15].

Different variants of composite materials require the diversification of testing techniques and the selection of those that will provide the most accurate measurement results conveniently and without generating excessive costs. In choosing these techniques, conditions are related not only to the specific properties of a particular composite material (such as light permeability in a selected range of wavelengths, conductivity, etc.) but also to broadly understand the operational conditions of the structure—its location, accessibility, or the possibility of temporary shutdown from operation [8]. It may be advantageous to perform the necessary measurement indirectly by determining the values of parameters associated with the monitored quantity. However, another methodologies may involve incorporating markers with state-dependent structural material properties into the composite’s structure, which are easily measurable: electrical, magnetic, or optical. Such an approach is typical for resistance strain gauges, magnetic methods—e.g., MRM, transmittance measurements, or reflectometry-like integral time-division multiplexing (TDM) with monitored sections of optical fiber lines incorporated into the structure.

In the operation of structural elements constructed from organic composites, a hazardous situation arises from the increase in deformations caused by temporary excessive loads. These loads may not easily lead to noticeable deformations or loss of stability, but they weaken the part’s microstructure and deterioration of the composite material, significantly reducing its strength [16]. This weakening can lead to catastrophic failure even within the design-specified load range. Preventing such a scenario necessitates monitoring the composite material’s structure or relatively monitoring the magnitude of deformations during operation.

A relatively convenient method for monitoring deformations involves using strain gauge technology (resistive tensiometry), wherein deformations of the surface of the tested element are transferred to a directly linked sensor, the resistance of which is dependent on the occurring deformation (elongation) on the surface [17,18,19]. This approach enables continuous deformation monitoring but relies on a constant power supply and intricate measurement circuits. While these inconveniences are manageable in laboratory conditions, they can effectively hinder the application of this method in field or mobile settings.

In the experiments presented below, we demonstrate that the MRM method (Magnetic Recording Method) described elsewhere [20,21] can be employed for the periodic monitoring of maximum deformations in structures made from nonmagnetic composite materials. The approach presented here, elaborated upon in subsequent paragraphs, combines the concept of a thin sensor (placed on the surface of the deforming element)—that is derived from resistive tensiometry—with the utilization of deformation-dependent permanent changes in the magnetization state of the sensor, which constitutes the essence of the MRM. In this implementation of the measurement, the sensor does not require a power supply. It simultaneously functions as a memory element, periodically providing information about the magnitudes of maximum deformations when read.

## 2. Materials

### 2.1. Laminates

A layered organic composite (laminate) was utilized as the material under investigation. It was produced from Nuvocryl FR60-100 polyester resin, a product of Mäder Group, (Vedène, France) and E-type glass fibers. Glass reinforcement was used in the form of 4-axis directional fabric with continuous fibers, Nuvofibre Quad 1200-150 brand (Aditya Birla Nuvo Ltd. in West Bengal, India), having a surface density of 1350 g/m^2^. The reinforcing material is a variation of stitched fabric with continuous fibers oriented at 0/+45/−45/90-degree angles, with a surface density of 300 g/m^2^ for each direction. The material is combined with the substrate as a glass veil with a 150 g/m^2^ surface density. Two layers of the mentioned composite reinforcement were used in the examined composite structure, symmetrically positioned relative to the laminate’s central plane and separated by a single layer of open-porous G-Flow mat. The G-Flow material is employed for technological reasons, facilitating the flow of liquid resin during laminate manufacturing using resin transfer molding (RTM) technology. The composite panels from which test samples were extracted were produced industrially by Astromal Ltd., Wilkowice, Poland. This material (and similar laminates utilizing similar raw materials) meets European fire safety requirements established for vehicles and is used for interior and exterior panels in railway carriages, trams, and buses.

The composite material samples were subjected to destructive testing of elastic-strength properties. The tests followed the PN-EN ISO 527-4 [22] standard on Type 2 samples, using an INSTRON 8850 (Instron Corporation, Norwood, MA, USA) servo-hydraulic testing machine equipped with Bluehill 3.0 software. A force transducer with a 25 kN range and a class 0.5 accuracy was employed. The machine operated in a climate-controlled laboratory at conditions of 23 °C and 50% relative humidity. Relative elongation was calculated based on measurements from electromechanical direct extensometers. The reference base of the extensometers was 50 mm (longitudinal strain) and 25 mm (transverse strain).

**Figure 1:**
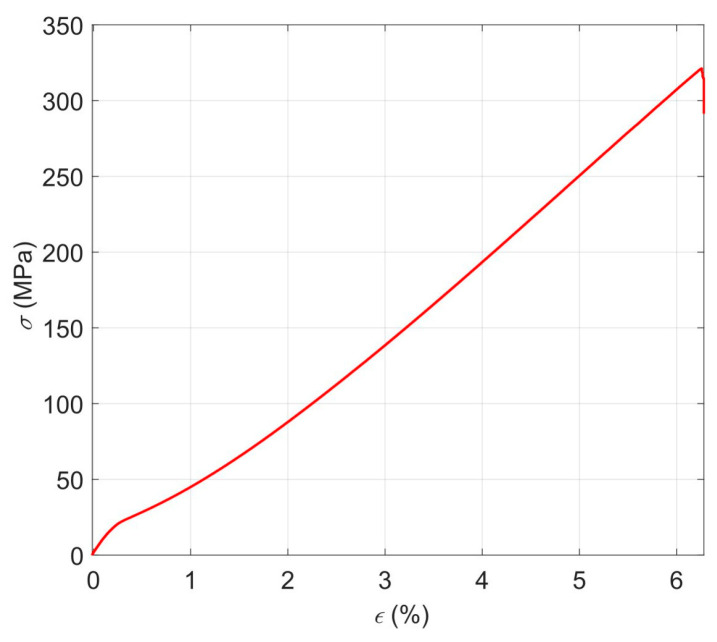
Stress–strain curve for the laminate.

The determined properties of the laminate are provided in Table 1 below. Figure 1 depicts the laminate’s representative stress–strain curve (σ(ε)).

### 2.2. Ferromagnetic Strips-Sensors

For fabricating strain sensors, ferromagnetic material was utilized as a rolled foil with a calibrated thickness of 50 μm, commercially available on the market. The foil was made of cold-rolled, deep-drawing grade low-carbon steel DC01. The mechanical and magnetic typical properties of the material are as follows: the 0.2% offset yield strength *R_P_*_02_ = 171 MPa, the tensile strength *R_m_* = 311 MPa, elongation *A*_80_ = 39.7%, magnetic induction *B*_50_ = 1.66 T, and *B*_150_ = 1.85 T. The foils, commonly used as calibration shims in machine building and precision mechanics, are conventionally offered in various thicknesses starting from 10 µm, with 10, 20, and 50 μm thickness increments. A foil with a thickness of 50 μm was selected based on earlier load-elongation measurements of variants with larger and smaller thicknesses to achieve deformations as closely aligned as possible with those of the laminate (a material with significantly lower elastic modulus than steel) without inducing excessive shear stresses in the adhesive layer bonding the sensor to the substrate. Simultaneously, optimizations were made to retain a substantial volume of ferromagnetic material to ensure favorable conditions for measuring changes in magnetization. From the foil, sensors were cut using a template and precise sheet metal shears, resulting in sizes of 10 mm × 100 mm.

### 2.3. Adhesive for Sensors

An epoxy adhesive was employed to affix the ferromagnetic strips (sensors) onto the surfaces of laminate samples. The adhesive was composed of epoxy resin EPIDIAN 6 and a stoichiometric addition of triethylenetetramine, which the resin manufacturer supplied as the curing agent.

### 2.4. GFRP Samples

The same variety of laminate samples was used for the testing as in the destructive tensile tests, namely Type 2 samples, according to EN ISO 527-4. In the region where the sensor was to be attached, the smooth surface of each sample was gently rubbed with corundum sandpaper with a grit size of 400. The area was meticulously cleaned and degreased with acetone and isopropanol. A mounting area for the sensor was demarcated on each sample symmetrically concerning all edges using self-adhesive masking tape.

**Figure 2:**
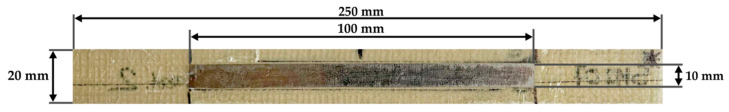
A photo of a GFRP sample with the strip sensor.

The surfaces of the sensor strips were cleaned and degreased with acetone and isopropanol. A freshly prepared epoxy adhesive layer was applied to the laminate and metal surfaces. The sensors were then affixed within the designated areas on the surfaces of the laminate samples and pressed onto the substrate using a 0.5 mm thick PTFE (polytetrafluoroethylene) film as a shim. A pressure of approximately 1 kg/cm^2^ was maintained for 24 h during the adhesive curing process. After this time, the masking tape markers were removed, the surfaces of the samples and sensors were cleaned with isopropanol, distinct markings were made on the sample surfaces, and the sensors were magnetized and preliminarily measured for the applied magnetic pattern. Figure 2 illustrates a sample with the strip sensor and its dimensions.

## 3. Methods

### 3.1. Magnetization Pattern Recording and Preliminary Measurements

**Figure 3:**
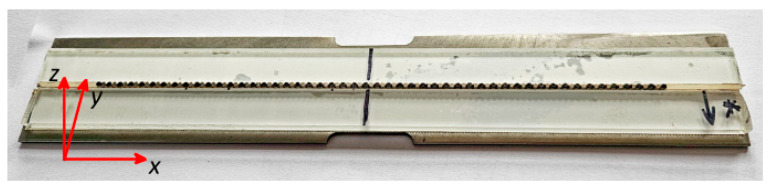
An array of permanent magnets as the magnetizing system. The samples are magnetized by moving the system along the *y*-axis.

Figure 3 shows a photo of a magnetizing system. The magnetizing system comprises 100 cylinder-shaped permanent magnets mounted over the ferromagnetic plate and surrounded with organic glass elements (Perspex). The height of a single cylindric permanent magnet is 5 mm, while the diameter is 2 mm. The pitch of the magnet array is equal to 2 mm, and it has alternating polarity (the first magnet has north facing up, the second magnet has south facing up, the third magnet has north facing up, etc.) Such a magnet arrangement creates a quasi-sinusoidal magnetization pattern in the steel strips. The recording process relies on manually moving the array with a speed of ca. 5 mm/s from the upper to the lower edge of the sample, parallel to the *y*-axis.

**Figure 4:**
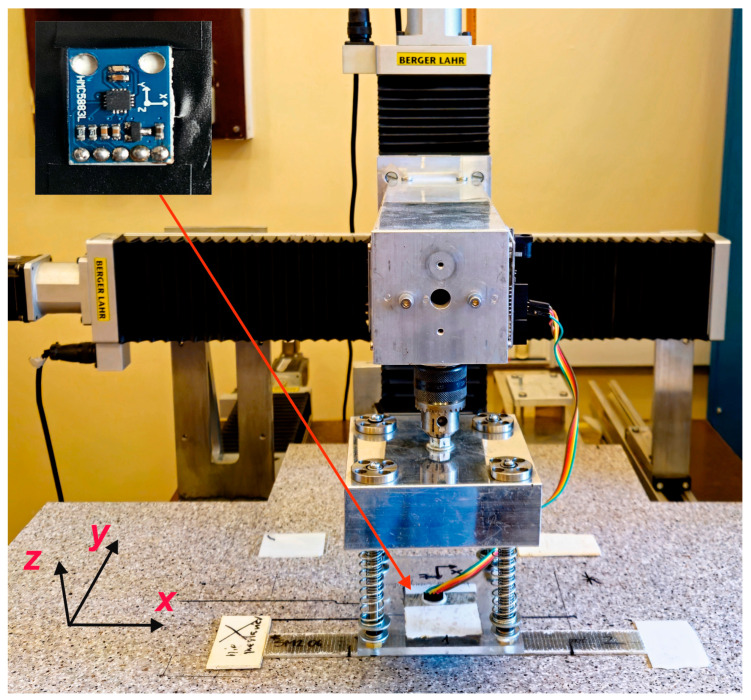
Positioning system with a sample and the HMC5883L magnetometer.

Following path recording, the residual magnetization was measured with an HMC5883L magnetometer (Honeywell, Plymouth, MA, USA) shifted with the positioning system (Figure 4). The magnetometer scanned the recorded magnetization patterns from the sample’s left to the right edge, line by line, parallel to the longitudinal axis. The signal was measured at each 0.5 mm. After one line was scanned to the end, the magnetometer was shifted to another line 1 mm apart along the latitudinal axis. Once the measurement was performed, the samples were subjected to the quasi-static tensile tests.

### 3.2. Quasi-Static Tensile Testing of Composite Samples

**Figure 5:**
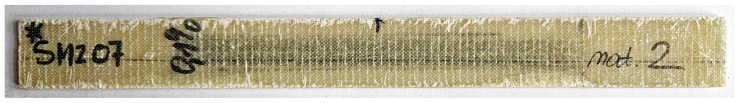
A photo of a sample after the stress test (the star mark was used to keep the same orientation of the sample during all tests).

Samples with sensor strips and recorded magnetization patterns were subjected to tensile testing using a universal INSTRON 3366 (Instron Corporation, Norwood, MA, USA) testing machine at a 2 mm/min speed. For each consecutive sample S01–S06, the relative strain was incrementally increased from approximately 0.1% to 1.8% with steps of about 0.3%. The samples were marked with signatures S01–S06 according to the increasing relative strain. The sample was unloaded at 100 mm/min after reaching the specified strain limit. A force transducer with a 10 kN range and a class 0.5 accuracy was used. The machine operated in a climate-controlled laboratory at conditions of 23 °C and 50% relative humidity. Relative elongation was calculated using measurements from the machine’s traverse displacement encoder. Within 48 h after deformation, residual magnetization patterns were measured once again. The measurement was performed analogously to the manner described in Section 3.1. An exemplary sample after the stress test is depicted in Figure 5.

## 4. Results

**Figure 6:**
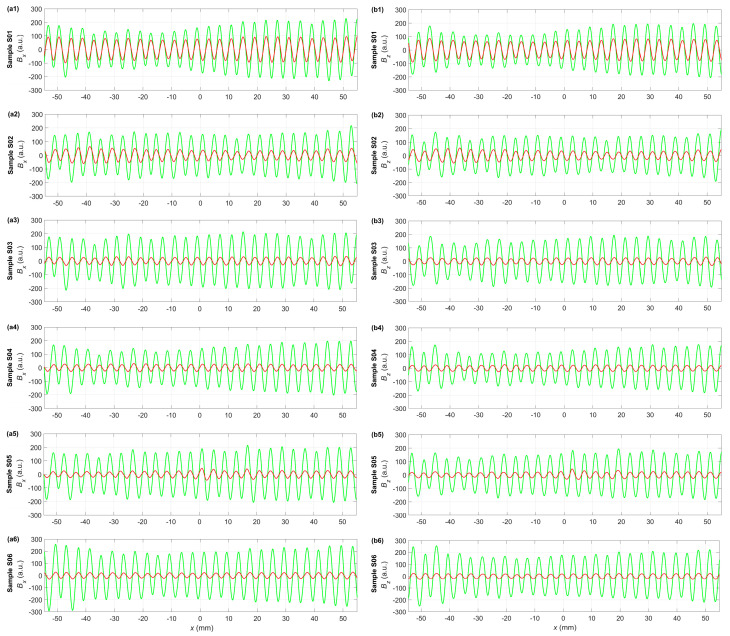
Mean residual magnetic field components before stress test (green curves) and after stress test (red curves): (**a1**–**a6**) *B_x_* for samples S01–S06, (**b1**–**b6**) *B_z_* for samples S01–S06.

**Figure 7:**
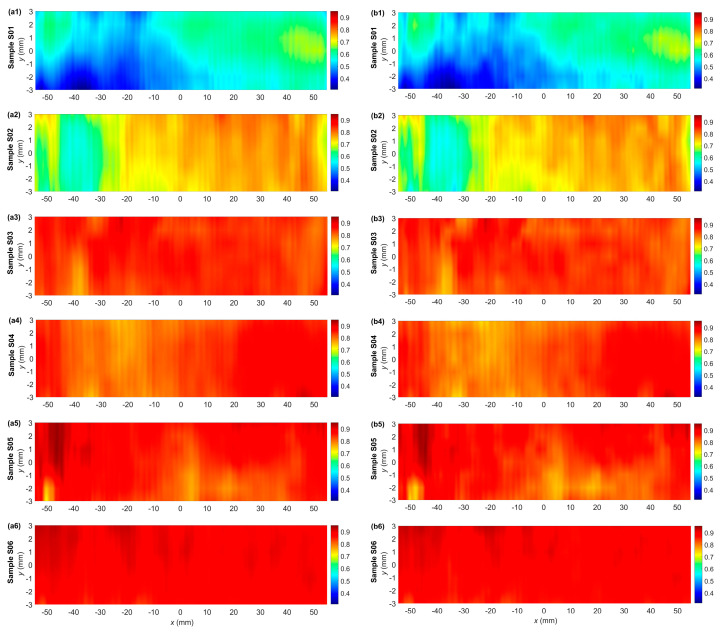
The plot of the 2D distribution of relative change in the RMS residual magnetization Δ*RMS_x_* (**a1**–**a6**) and Δ*RMS_z_* (**b1**–**b6**) measured in case of samples S01–S06.

The experiment measured three residual magnetization components of each sample: *B_x_*, *B_y_*, and *B_z_*. Component *B_y_* was excluded from the data analysis because of its low value and minor significance. The signals measured for each sample were averaged to give a preliminary insight into the sample’s condition. Figure 6 encapsulates the two selected averaged magnetization components as a function of the sensor position along the sample’s longitudinal axis. However, it only provides fundamental information about the signal trends in the samples. Thus, an additional data processing algorithm based on the calculation of sine wave characteristic parameters gives a more comprehensive overview of the changes in magnetization patterns. This study opts for the RMS (root mean square) values for material condition assessment (Figure 7).

### 4.1. Analysis of the Signal Obtained along the Center Line

The averaged magnetization patterns allow for an immediate presentation of the method’s applicability for the composites. Calculations were performed for each sample’s *B_x_* and *B_z_* magnetization components before and after the stress tests.

The formula given below stands for the average value of the residual magnetization:

(1) $$B_{cm}=\frac{1}{n}\sum_{i=1}^{n}B_{ci}$$

where *c*—the x, y, or z component of the residual magnetic field, *m* = 1 for the residual magnetization measured before the stress test, *m* = 2 for the residual magnetization measured after the stress test, *i*—the number of the magnetization pattern, and *n*—the total number of subsequent magnetization patterns included in the calculations.

Figure 6 presents changes in the averaged amplitude of the *B_x_* (a1–a6) and *B_z_* (b1–b6) components plotted against the sensor position along the longitudinal axis. The averaged signal before the stress test is green, and the one after the test is red. Independent of the component chosen, the most remarkable feature of the signal after load is that its amplitude decrease corresponds with the increasingly stretched samples. There are no evident frequency variations. The averaged signals in Figure 6 show the amplitude variations caused by the applied stress. For this reason, a more comprehensive analysis is required, including a complete set of the measured signals after preprocessing.

### 4.2. Analysis of the Signals Measured on the Rectangular Grid

The RMS (root mean square) calculations were performed to analyze the measurement results thoroughly. The RMS was calculated for sequential signal samples extracted from a window. The window length equals the number of samples within one signal period. The window was shifted one sample at a time along the scanning line parallel to the latitudinal axis. Once the RMS calculation for one scanning line was finished, the window moved along the latitudinal axis to the subsequent line, and the process was repeated.

The following formula calculates the RMS value for the residual magnetization:

(2) $${RMS}_{cm}=\sqrt{\frac{1}{n}\sum_{i}B_{cm}^{2}(i)}$$

where *c* for *x*, *y*, or *z* component of the residual magnetic field, *m* = 1 for the residual magnetization measured before the stress test, *m* = 2 for the residual magnetization measured after the stress test, *n*—number of samples per period.

Ultimately, the relative change in the RMS value is given by the following:

(3) $${∆RMS}_{c}=\frac{{RMS}_{c1}}{{RMS}_{c2}}$$

Figure 7 illustrates the amplitude variations in the RMS values of the B_x_ component (Δ*RMS_x_*) and *B_z_* component (Δ*RMS_z_*) measurements. As is evident from plots a1 to a3 in Figure 7, the Δ*RMS_x_* for the samples S01–S03 grows parallel with the increasing strain level. A drop in the parameter value is evident for sample S04, which has a strain level corresponding to the yield point (Figure 7(a4)). For the last two samples beyond the yield point, S05 and S06, the Δ*RMS_x_* goes up again. The changes in the Δ*RMS_z_*, depicted in Figure 7(b1–b6), are generally analogous to those observed for the *B_x_* component.

### 4.3. Statistical Analysis

**Figure 8:**
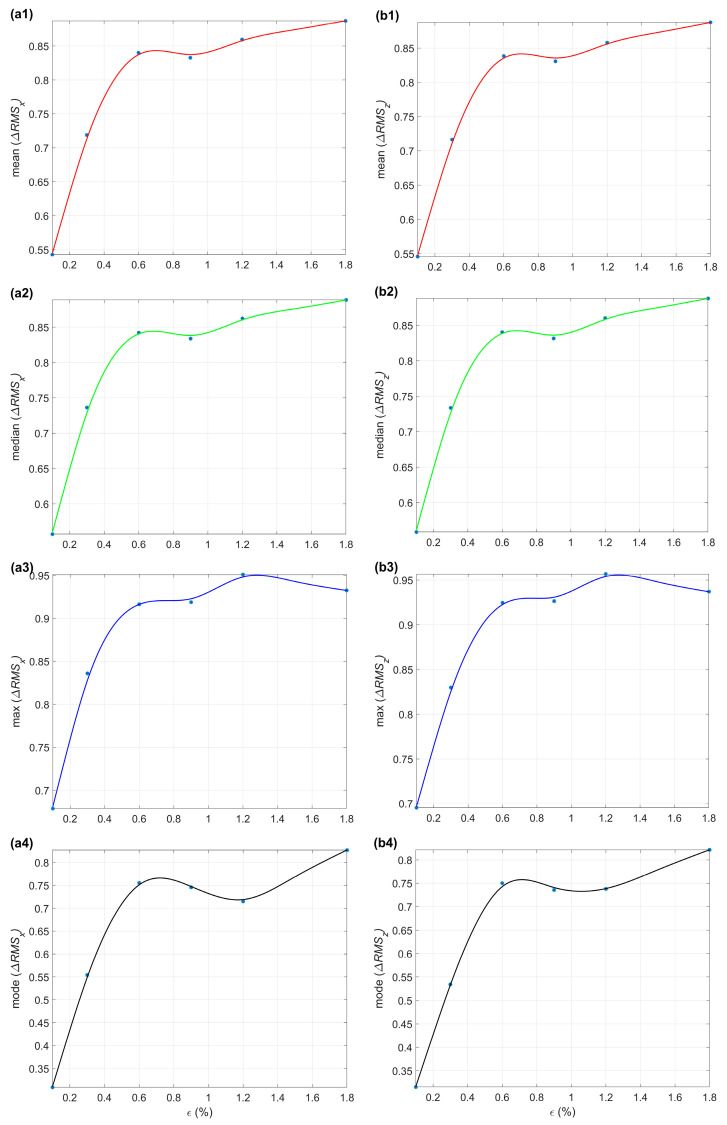
Statistical parameters (mean—(**a1**,**b1**); median—(**a2**,**b2**); maximum—(**a3**,**b3**); mode—(**a4**,**b4**)) calculated for the relative change in the RMS residual magnetization Δ*RMS_x_* (**a1**–**a4**) and Δ*RMS_z_* (**b1**–**b4**).

The statistical analysis aims to deliver an unambiguous overview of the general signal trends in the subsequent samples. This study considers four parameters of the Δ*RMS_x_* and Δ*RMS_z_*, i.e., the mean, median, maximum, and mode. The statistical analysis results for the Δ*RMS_x_* are depicted in Figure 8(a1–a4), while for the Δ*RMS_z_*_,_ in Figure 8(b1–b4).

For the samples S01–S03 from the elastic region (Figure 8(a1,b1)), the mean value of the Δ*RMS_x_* or Δ*RMS_z_* increases as the strain level rises. Subsequently, the curve drops down for the S04 sample loaded to the yield point. In the plastic region, the curve values for the samples S05 and S06 increase anew. The same situation occurs for the median of the Δ*RMS_x_* or Δ*RMS_z_* (Figure 8(a2,b2)). Regarding the maximum value of the Δ*RMS_x_* or Δ*RMS_z_* curves (Figure 8(a3,b3)), it can be stated that the curves grow sharply up to sample S03, then slow down at sample S04, and increase up to sample S05. The mode curves (Figure 8(a4,b4)) have a slightly different course. In the initial section, they increase approximately linearly up to sample S03. Then, they decrease and maintain such a course until sample S05. While approaching S06, the curves grow sharply. The results confirm that the method allows for unambiguously determining the applied load, especially in the elastic region.

It is possible to explain [23] the changes in the magnetization pattern introduced in the sample volume caused by the stresses in the material and the accompanying deformations (of elastic nature), and by the tendency of the magnetic domains that make up the magnetization pattern to reach the state with the lowest possible free energy. Before magnetization, the volume of the material of interest is in a stable energy state with a specific minimum free energy value. As a result of magnetization, the energy state of the material changes (the energy of the analyzed sample volume increases), and it reaches a new metastable level. At the scale of the crystal structure of the ferromagnetic material, this is achieved by the displacement and growth of the surface of the magnetic domain boundaries (Neel and Bloch walls) in accordance with the magnetic field lines, which force the magnetization pattern of the sample. The stabilization of domain boundaries in metastable positions occurs due to blocking them on defects in the material’s crystal structure, such as vacancies, dislocations, or non-ferromagnetic inclusions. The deformation of the crystal lattice, eventually caused by mechanical stresses, deactivates the places of immobilization of the domain boundaries to a greater or lesser extent and enables the spontaneous change of their position towards other metastable states with lower energy. This can be observed macroscopically as the changes in the sample’s magnetization pattern described in this work.

## 5. Conclusions

The study employed the Magnetic Recording Method, generally destined for ferromagnetic materials, to inspect tensile stress-loaded GFRP components. The research presented in this paper is the first attempt to apply the Magnetic Recording Method to a nonmagnetic structure. Based on the results of the study, the following conclusions can be drawn:

- Using metal ferromagnetic strip sensors, the Magnetic Recording Method may be successfully employed to evaluate the load of nonmagnetic materials, such as GFRPs.
- The quasi-sinusoidal magnetization patterns recorded on the strip sensors vary their characteristic parameters with the successively strained samples. In contrast with the study results for the metal samples [20,21], only amplitude is utilizable for the unequivocal condition assessment of the GFRPs. The changes in the frequency are barely visible and thus cannot be used as a criterion.
- The calculated relative changes in the RMS value and statistical parameters enable an evaluation of the variations in the magnetization patterns caused by tensile stress. Due to the monotonicity of statistical parameters’ curves, the condition of each sample can be assessed unambiguously for the strain up to 0.6%. The precise identification of the stress level above this limit is no longer possible, especially near the yield point. The most clear-cut results can be obtained from the median value plot. This issue will be investigated in the future works.
- To sum up, the difference is that in the case of ferromagnetic structures [20,21], no additional tape is necessary because the material itself can be used as a sensor element. Concerning non-ferromagnetic structures, it is necessary to connect the sensor with a tape made of ferromagnetic material to the tested structure. This situation means that, as in the case of ferromagnetics, it is impossible to test the internal stress occurring in the material, and only the stress on the surface of the tested element is monitored. Furthermore, the use of glue or other binders may cause measurement errors, especially in the case of partial detachment. Despite these problems, the proposed method allows for the obtainment of information about the load history of the tested structure, which, in the case of other methods, requires complex measurement systems with continuous recording.

In this study, we have presented the feasibility of physically implementing the “magnetic tensometry” method and the prospect of its practical application in monitoring constructions. Further research, which we deem necessary, will allow for the optimization of the selection of the magnetic strip material based on the type of material in the monitored structure and the environmental operating conditions. The choice of bonding agents for the magnetic strip with the monitored structure, tailored to the adhesive properties of the combined materials, is also of significant importance.

We believe that our method will be suitable for studying composite materials utilizing a wide range of polymer matrices and various reinforcing fibers (e.g., carbon, basalt, aramid). This assertion is justified by the similar elastic modulus ratio between the material of the “magnetic tensometer” and most composite materials. However, it will require further studies.

## Figures and Tables

**Table 1:** Laminate properties.

Property	Unit	Mean Value	Std. Deviation	Description
*R_m_*	MPa	254	12	Tensile strength
*E_sε_*	GPa	17.4	0.935	Young modulus
*ν_sε_*	-	0.26	0.071	Poisson’s ratio

## Data Availability

Data available on request due to large size and complicate structure.
